# Clinical manifestations and visual outcomes associated with ocular toxoplasmosis in a Brazilian population

**DOI:** 10.1038/s41598-021-82830-z

**Published:** 2021-02-04

**Authors:** Sigrid Arruda, Barbara R. Vieira, Denny M. Garcia, Michelle Araújo, Milena Simões, Renata Moreto, Murilo W. Rodrigues, Rubens Belfort, Justine R. Smith, João M. Furtado

**Affiliations:** 1 Division of Ophthalmology, Ribeirão Preto Medical School, University of São Paulo, Avenida Bandeirantes 3900, Ribeirão Preto, São Paulo 14049-900 Brazil; 2 Department of Ophthalmology, Federal University of São Paulo, São Paulo, Brazil; 3 College of Medicine and Public Health, Flinders University, Adelaide, South Australia Australia

**Keywords:** Signs and symptoms, Eye manifestations

## Abstract

Ocular toxoplasmosis is the leading cause of posterior uveitis worldwide. We conducted an observational study of 262 consecutive individuals (n = 344 eyes) with ocular toxoplasmosis who were followed over a 34-month period. Most subjects were *T. gondii* IgG + /IgM- (n = 242; 92.4%; 317 eyes), and 140 eyes (40.7%) had active lesions. For eyes in which retinal lesions were active at recruitment and best-corrected visual acuity (BCVA) could be measured (n = 133), 21.0% (n = 28) remained blind (BCVA below 20/400) after inflammation resolved. In these eyes, atypical ocular toxoplasmosis (OR 4.99; 95% CI 1.14–22.85; *p* = 0.0330), macular lesion (OR 9.95; 95% CI 2.45–47.15; *p* = 0.0019) and any complication (OR 10.26; 95% CI 3.82–30.67; *p* < 0.0001) were associated with BCVA below 20/200. For eyes with only inactive lesions at recruitment and BCVA measured (n = 178), 28.1% (n = 50) were blind. In these eyes, having at least one lesion larger than one disc-diameter (OR 6.30; 95% CI 2.28–22.46; *p* = 0.0013) and macular lesion (OR 5.69; 95% CI 2.53–13.54; *p* < 0.0001) were associated with BCVA below 20/200. Older age (OR 1.02; 95% CI 1.00–1.05; *p* = 0.0493) and active disease at presentation (OR 4.74; 95% CI 1.95–12.91; *p* = 0.0011) were associated with recurrences. Additional clinical attention should be directed towards patients with risk factors for poor visual outcome.

## Introduction

The Apicomplexan protozoan, *Toxoplasma gondii*, infects an estimated 25% to 30% of the human population^1^. Although the infection is usually asymptomatic in otherwise healthy humans, multiple clinical manifestations of acute and chronic infection are widely described, and ocular toxoplasmosis is the most common manifestation in chronically infected subjects^2, 3^. Ocular toxoplasmosis almost always presents as necrotizing retinitis that is associated with inflammation in adjacent ocular tissues^3^, and the condition is one of the leading causes of posterior uveitis worldwide^4^. In Latin America, where the prevalence of *T. gondii* infection is relatively high^5^, ocular toxoplasmosis is the most common form of uveitis^6, 7^, and also a major cause of childhood blindness^8^.

While many individuals presenting ocular toxoplasmosis have normal visual acuity and a satisfactory vision-related quality of life^9^, ocular toxoplasmosis is frequently a sight-threatening condition. Several hospital-based studies describe visual impairment in up to one in four affected eyes, most often associated with central retinal lesions^10, 11^. Since the retinal tissue does not regenerate, even individuals who receive appropriate antimicrobial and anti-inflammatory drug treatment may suffer irreversible blindness (best-corrected visual acuity less than 20/400). Human hosts cannot eliminate *T. gondii*, and therefore persons with ocular toxoplasmosis remain at risk of reactivation of the disease, and ocular complications may occur years after the initial infection^2, 3^.

The majority of data that describe risk factors for the development of recurrent ocular toxoplasmosis and visual impairment caused by the disease come from retrospective studies conducted on European and North America populations^10^. In these regions of the world, ocular toxoplasmosis is believed to be less severe than in South and Central America^4^. Understanding which individuals have an increased risk of developing recurrent disease and vision loss is important for the ophthalmologist. In describing a large and prospectively studied Brazilian patient group, we aimed to determine the clinical manifestations and risk factors for poor visual outcomes of ocular toxoplasmosis in Latin America.

## Results

Clinical data were collected for 262 individuals (n = 344 eyes), who presented to the Ribeirão Preto General Hospital Uveitis Clinic with active or inactive ocular toxoplasmosis during the 34-month study period commencing January 14th, 2015 (Table 1). Mean follow-up was 12.9 months (standard deviation, 8.4 months; median, 11.9 months; range, 0–30.1 months). The study included 139 women (53.1%) and 123 men (46.9%). Within the group, 47.3% individuals (n = 124) considered themselves multiracial, 38.2% (n = 100) reported Caucasian ethnicity, 13.4% (n = 35) reported African-Brazilian ethnicity, and 1.1% (n = 3) did not report their ethnic background (Table 1). Age at inclusion was under 18 years in 11.1% of the persons (n = 29), 18–64 years in 79.8% (n = 209) and over 64 years in 9.2% (n = 24). Within the group of 262 persons, 3.8% (n = 10) were infected with human immunodeficiency virus (HIV).

**Table 1:** Characteristics of the participants (n = 262 subjects).

Variable	N (%)
**Sex:**	
Women	139 (53.1)
Men	123 (46.9)
**Age (years):**	
≤ 17	29 (11.1)
18–64	209 (79.8)
≥ 65	24 (9.2)
**Self-reported ethnicity**:	
Multiracial	124 (47.3)
Caucasian	100 (38.2)
African-Brazilian	35 (13.4)
Not reported	3 (1.1)
**Form of ocular toxoplasmosis:**	
Primary active	48 (18.3)
Recurrent active	85 (32.4)
Inactive	129 (49.3)
**Type of ocular toxoplasmosis:**	
Typical	253 (96.6)
Atypical	9 (3.4)
**Mode of infection**:	
Congenital	24 (9.1)
Acquired	13 (5.0)
Unknown	225 (85.9)
***T. gondii serology***:	
IgG + IgM +	20 (7.6)
IgG + IgM-	242 (92.4)
**Previous treatment**:	
Never treated	49 (18.7)
Sulfamethoxazole and Trimethoprim	151 (57.6)
Sulfadiazine and Pyrimethamine	39 (14.9)
Unknown or other	23 (8.8)

For 50.7% (n = 133; 167 eyes) of the participants, ocular toxoplasmosis was active, and most often recurrent (n = 85; 115 eyes); and in 49.3% (n = 129; 177 eyes) of the participants, ocular toxoplasmosis was inactive (Table 1). Disease had a typical clinical appearance in most individuals (96.6%, n = 253; 330 eyes), and the mode of infection usually could not be determined in 85.9% (n = 225; 286 eyes). Just 7.6% of subjects (n = 20; 27 eyes) had serological evidence of acute infection (serum *T. gondii* IgG-positive and IgM-positive).

At presentation, 48.0% (n = 165) of the 344 affected eyes had a single retinal lesion, while 147 eyes (42.7%) had two to four lesions, and 32 eyes (9.3%) had five or more lesions (Table 2). Approximately half of the eyes had only peripheral retinal lesions (50.9%, n = 175), while the other half (49.1%, n = 169) had at least one lesion at the posterior pole. Most retinal lesions were measured greater than one disc-diameter (81.7%, n = 281). The lesions were active in 41.1% of eyes (n = 141), and 83.7% of eyes (n = 288) contained scars.

**Table 2:** Number, location, size of the retinal lesions and associated ocular findings at presentation (n = 344 eyes).

Variables	N (%)
**Number of lesions**:	
1	165 (48.0)
2–4	147 (42.7)
≥ 5	32 (9.3)
**Location of retinal lesion(s)**:	
Periphery	175 (50.9)
Posterior pole: macula	64 (18.6)
Posterior pole: outside macula	60 (17.4)
Periphery and posterior pole	45 (13.1)
**Size of the lesions**:	
≤ 1 DD	63 (18.3)
> 1 DD	281 (81.7)
Ocular findings*	
Retinal scar(s)	288 (83.7)
Active retinal lesion(s)	141 (41.0)
Vitritis	77 (22.4)
Vasculitis	15 (4.4)
Papillitis	3 (0.9)
Optic atrophy	3 (0.9)
Epiretinal membrane	2 (0.6)
Serous retinal detachment	2 (0.6)
Other**	4 (1.2)

DD = disc-diameter. *More than one posterior segment finding could be present per eye; **Macular edema (n = 1), choroidal neovascularization (n = 1), intraretinal hemorrhage (n = 1) and branch retinal vein occlusion (n = 1).

For the eyes in which retinal lesions were active at recruitment and best-corrected visual acuity could be measured (n = 133), 35.3% (n = 47) had normal acuity, 12.8% (n = 17) had acuities from 20/50 to 20/63, 18.1% (n = 24) had acuities from 20/63 to 20/160, 3.0% (n = 4) had acuities from 20/200 to 20/400, and 30.8% (n = 41) had visual acuities below 20/400 (Table 3). After the activity resolved in the 133 eyes, 56.4% (n = 75) had normal vision, and 21.0% (n = 28) were blind. Among these eyes, atypical ocular toxoplasmosis (odds ratio [OR]: 4.99; 95% confidence intervals [CI]: 1.14–22.85; *p* = 0.0330), macular lesion (OR 9.95; 95% CI 2.45–47.15; *p* = 0.0019) and development of any complication (OR 10.26; 95% CI 3.82–30.67; *p* < 0.0001) were associated with poor visual prognosis (Table 4). Best-corrected visual acuity could not be measured in 25 eyes (1 with an active lesion and 24 with inactive lesions) of 15 preverbal children.

**Table 3:** Initial and final best-corrected visual acuity of eyes with ocular toxoplasmosis (n = 311 eyes*).

Best-corrected visual acuity	At presentation	Final
	N (%)	N (%)
**Eyes with active lesions at presentation (n = 133 eyes)**		
Normal vision (≥ 20/40)	47 (35.3)	75 (56.4)
Mild visual impairment (20/50 to 20/63)	17 (12.8)	12 (9.0)
Moderate visual impairment (< 20/63 to 20/160)	24 (18.1)	11 (8.3)
Severe visual impairment (≤ 20/200 to 20/400)	4 (3.0)	7 (5.3)
Blindness (< 20/400)	41 (30.8)	28 (21.0)
**Eyes with inactive lesions at presentation (n = 178 eyes)**		
Normal vision (≥ 20/40)	81 (45.5)	
Mild visual impairment (20/50 to 20/63)	17 (9.5)	
Moderate visual impairment (< 20/63 to 20/160)	14 (7.9)	
Severe visual impairment (≤ 20/200 to 20/400)	16 (9.0)	
Blindness (< 20/400)	50 (28.1)	

*Best-corrected visual acuity could not be measured in 25 eyes (1 with an active lesion and 24 with inactive lesions) as subjects were preverbal children. For those with inactive lesions visual acuity at presentation = final visual acuity. Seven adults (8 eyes) were not followed until inactivation of ocular disease, and were removed from this analysis.

**Table 4:** Multivariate binary logistic regression of variables associated with poor visual prognosis in eyes with active ocular toxoplasmosis at presentation (final best-corrected visual acuity ≤ 20/200; n = 133 eyes).

Variable	Odds ratio (95% CI)	*p* value
Older age (≥ 65 years old)	0.99 (0.96–1.02)	0.47
Male sex	0.57 (0.21–1.48)	0.25
Atypical ocular toxoplasmosis	4.99 (1.14–22.85)	**0.0330**
Presence of any complication	10.26 (3.82–30.67)	** < .0001**
Reactivation during follow-up	0.48 (0.13–1.63)	0.26
Size of the lesion (> 1 DD)	1.02 (0.25–5.24)	0.98
Location (macula)	9.95 (2.45–47.15)	**0.0019**

DD = disc-diameter; CI = confidence interval.

For the eyes in which there were only inactive lesions at recruitment and best-corrected visual acuity could be measured (n = 178), 45.5% (n = 81) had normal acuity, while 28.1% (n = 50) had visual acuities below 20/400 (Table 3). In these 178 eyes, the presence of at least one lesion that measured greater than one disc-diameter in size (OR 6.30; 95% CI 2.28–22.46; *p* = 0.0013) and macular lesion (OR 5.69; 95% CI 2.53–13.54; *p* < 0.0001) were associated with a poor visual prognosis (Table 5).

**Table 5:** Multivariate binary logistic regression of variables associated with poor visual prognosis in eyes with inactive ocular toxoplasmosis at presentation (final best-corrected visual acuity ≤ 20/200; n = 178 eyes).

Variable	Odds ratio (95% CI)	*p* value
Older age (≥ 65 years old)	1.01 (0.99–1.03)	0.16
Male sex	1.07 (0.53–2.16)	0.84
Presence of any complication	1.96 (0.61–6.21)	0.25
Size of the lesion (> 1 DD)	6.30 (2.28–22.46)	**0.0013**
Location (macula)	5.69 (2.53–13.54)	** < .0001**

DD = disc-diameter; CI = confidence interval.

Considering the visual acuity of the better-seeing eye in those with bilateral retinal lesions (either active or inactive ocular toxoplasmosis) and with final best-corrected visual acuity measured (n = 71 subjects), 73.2% (n = 52) had normal acuity, 9.9% (n = 7) had acuities from 20/50 to 20/63, 2.8% (n = 2) had acuities from 20/63 to 20/160, 5.6% (n = 4) had acuities from 20/200 to 20/400, and 8.5% (n = 6) had acuities below 20/400, rendering the individual bilaterally blind.

Older age (OR 1.02; 95% CI 1.00–1.05; *p* = 0.0493) and active ocular toxoplasmosis at presentation (OR 4.74; 95% CI 1.95–12.91; *p* = 0.0011) were the only risk factors associated with recurrence during follow-up (Table 6). Considering just the eyes that were followed over time (n = 292), we observed 28 episodes of reactivation during the follow-up period, with 21 of these reactivations occurring in eyes with active retinal lesions at enrollment. This equated to 0.091 (0.060 – 0.131, 95% CI) reactivations per eye-year across eyes presenting with either active or inactive lesions, and 0.172 (0.106 – 0.262, 95% CI) reactivations per eye-year for those eyes with active lesions at presentation.

**Table 6:** Multivariate binary logistic regression of variables associated with reactivation of ocular toxoplasmosis (n = 292 eyes).

Variable	Odds ratio (95% CI)	*p*
Older age (≥ 65 years old)	1.02 (1.00–1.05)	**0.0493**
Number of Lesions (> 1)	0.68 (0.28–1.62)	0.38
Male sex	0.86 (0.37–1.94)	0.71
Active disease at presentation	4.74 (1.95–12.91)	**0.0011**
Size of the lesion (> 1 DD)	1.99 (0.61–9.03)	0.30

DD = disc-diameter; CI = confidence interval.

## Discussion

In this study of a large series of 262 Brazilian women and men who were followed prospectively for ocular toxoplasmosis, we observed permanent vision loss in approximately one-half of the eyes, with one in four eyes blind, and with six patients bilaterally blind. Among those eyes with active retinitis at presentation, we identified three clinical features that were associated with a final best-corrected visual acuity equal to or below 20/200: atypical ocular toxoplasmosis, the presence of a macula lesion, and the development of any ocular complication during follow up. For eyes that presented with inactive retinal scars, we found lesions in the macula and lesions measuring over one disc-diameter to be associated with this severe level of vision impairment. Older age and the presence of an active retinal lesion at presentation were associated with higher risk of disease recurrence, which may progress any vision loss.

Several other investigator teams have reported associations between characteristics of active ocular toxoplasmosis and severe vision impairment that are largely consistent with our findings in this larger group that also includes patients with inactive disease. One team, also from Brazil, prospectively studied a group of 230 subjects, and linked the same level of vision loss with location of the retinal lesion, presence of posterior eye complications and recurrence during follow-up^11^. Another team, based in The Netherlands, retrospectively evaluated a group of 154 consecutive patients, and associated size and location of the retinal lesion, as well as primary ocular toxoplasmosis and congenital toxoplasmosis, with visual acuity of 20/200 or worse. We did not assess the patient group for an association with the mode of infection (congenital or postnatally acquired), since the mode is seldom known with certainty.

Our study revealed that those individuals with active toxoplasmic retinal lesions at recruitment were at a higher risk of recurrence when compared to persons with inactive lesions (*p* = 0.001). This is in keeping with previous work describing recurrence patterns of ocular toxoplasmosis in a group of Dutch patients, which showed that recurrence risk was highest immediately after an episode of active disease and reduced with an increasing disease-free interval^12^. The alternate daily use of trimethoprim and sulfamethoxazole for one year after an active episode of ocular toxoplasmosis was first described by Brazilian ophthalmologists in 2002 as an option to reduce the risk of recurrences^13^. Subsequent studies that were also conducted in Brazil supported the preventive effect of the approach^14^, and showed that this effect was sustained over multiple years of follow-up^15, 16^.

Although the prophylactic use of trimethoprim and sulfamethoxazole in patients with ocular toxoplasmosis has become popular in Brazil^17^, the value of this therapy in non-endemic areas is less clear. While our study and previous reports suggest that patients at a higher risk of recurrence are those with a recent episode of active ocular toxoplasmosis, most of these people do not develop ocular recurrences within a short time frame. For this reason, we recommend preventive treatment be considered only for those patients with an increased risk of vision loss if their disease recurs. Recognizing that macular lesions are associated with a poor visual prognosis, and that active lesions frequently develop adjacent to retinochoroidal scars, an individualized approach may be taken to prophylactic drug therapy following recently active ocular toxoplasmosis.

Strengths of our study include the substantial number of prospectively enrolled subjects in an endemic area, the standardized process of data collection by a small number of specialized researchers, and the use of polymerase chain reaction (PCR) on intraocular fluid samples to confirm those cases of atypical toxoplasmosis. One limitation of our work, which is common to all studies on ocular toxoplasmosis, is the need to rely on clinical assessment and serological testing to make the diagnosis, since retinal biopsy poses an unacceptable risk to vision. Another limitation relates to selection bias: this study was conducted at a tertiary referral hospital clinic, and thus the percentage of patients who experienced severe visual impairment was probably higher than in the total population with ocular toxoplasmosis, since people with mild or no ocular symptoms would be unlikely to seek medical evaluation.

Our findings in this large patient group indicate that a significant percentage of eyes with ocular toxoplasmosis suffer vision loss, and suggest that particular clinical attention should be given to those with atypical manifestations of active disease, macular lesions and/or ocular complications, due to the increased risk of poor visual outcomes.

## Materials and methods

All persons with the diagnosis of active or inactive ocular toxoplasmosis, who attended the Uveitis Clinic at Ribeirão Preto General Hospital (Ribeirão Preto, São Paulo, Brazil) consecutively from January 14th, 2015 to November 16th, 2017 were enrolled in this observational study. The research protocol followed the principles of the Declaration of Helsinki, and was approved by the Ethics Committee in Human Research at Ribeirão Preto General Hospital (approval number: 46015415.2.0000.5440). All subjects agreed to participate in the study and signed a consent form approved by the Committee; children were only included with the consent of their legal guardian.

Study subjects had a comprehensive ophthalmic examination, including measurement of best-corrected visual acuity, Goldmann applanation tonometry and dilated fundoscopy. Retinal lesions were photographed with either the Canon CR-2 non-mydriatic retinal camera (Melville, NY) or the Topcon TRC-50DX mydriatic retinal camera (Oakland, NJ), unless the ocular media were too densely opacified for clear imaging. Ocular toxoplasmosis was diagnosed by a uveitis-specialized ophthalmologist (JMF) based on the observation of active or inactive toxoplasmic retinal lesions, and the presence of serum immunoglobulin (Ig)M and/or IgG directed against *T. gondii*. In atypical presentations, intraocular fluid was tested for *T. gondii* DNA by PCR, and selected other investigations were ordered depending on the differential diagnosis. Serological testing for HIV was routinely performed.

Patients with active retinal lesions were treated with an oral course of either trimethoprim and sulfamethoxazole, or sulfadiazine and pyrimethamine, plus oral prednisone. In a small number of cases, when individuals were unable to use sulfamethoxazole and sulfadiazine, oral or intravitreal clindamycin was used. The patients were re-evaluated 14 days and 45–60 days after presentation, unless the clinical situation required more frequent assessment. Patients with inactive retinal lesions were followed on a case-by-case analysis.

Ocular toxoplasmosis was classified according to the form of presentation (primary active, recurrent active or inactive) and the mode of infection (congenital, acquired or unknown)^10^. Clinical information that was collected in addition to the descriptors of ocular toxoplasmosis included patient sex, age at recruitment and self-reported ethnicity.

Toxoplasmic retinal lesions were designated “active” when characterized by fluffy white retinitis and vitritis, with or without with retinal scars, and “inactive”, when characterized by well-defined atrophic retinochoroidal scar(s) without retinitis or vitritis^2, 3^. The lesions were described according to location (central retina [macular and non-macular, posterior pole] and peripheral retina), number and size, which was estimated by comparison to the optic disc (in disc-diameters). A presentation was considered “atypical” if there were multiple or extensive (greater than 10 disc-diameters in size) retinal lesions^2^. Ocular complications were recorded, including persistent vitreous opacities (present 3 months after resolution of active lesion(s)), raised intraocular pressure, glaucoma, posterior synechiae, cataract, epiretinal membrane, macular edema, macular hole, retinal tear, retinal detachment, retinal vascular occlusion, retinal and choroidal neovascularization, vitreous hemorrhage, optic atrophy and phthisis bulbi.

The descriptive results were presented as frequencies and percentages. The 95% CI were calculated for the estimated incidence rate of reactivation. Multivariate logistic regression was used to assess the risk factors associated with poor visual prognosis and reactivation of ocular toxoplasmosis. Statistical analyses were performed using R software (version 3.6.3; The R Foundation for Statistical Computing). A p-value of less than 0.05 was taken to indicate a significant difference.
